# Supplementary material for: Turn-timing in conversations between autistic adults: Typical short-gap transitions are preferred, but not achieved instantly
Source: PLoS One. 2023 Apr 6;18(4):e0284029. doi: 10.1371/journal.pone.0284029 (PMC10079028; doi:10.1371/journal.pone.0284029)
Supplement: S1 Table — (PDF) [file pone.0284029.s001.pdf]

S1 Table. Summary of dialogue duration, number of IPUs and transition types, by group.

Group	Mean dialogue duration (SD)	Total IPUs	Total turn transitions	Total silent gaps (% of all transitions)	Total between-overlaps (% of all transitions)	Total within-overlaps (% of all transitions)
ASD	14’ 37’’ (7’ 12’’)	6211	1841	1168 (63.4 %)	388 (21.1 %)	285 (15.5 %)
CTR	26’ 01’’ (14’ 35’’)	12121	3827	2250 (58.8 %)	938 (24.5 %)	639 (16.7 %)
Total	20’ 19’’ (12’ 32’’)	18332	5668	3418 (60.3 %)	1326 (23.4 %)	924 (16.3 %)
