# Supplementary figures and images for: Turn-timing in conversations between autistic adults: Typical short-gap transitions are preferred, but not achieved instantly
Source: PLoS One. 2023 Apr 6;18(4):e0284029. doi: 10.1371/journal.pone.0284029 (PMC10079028; doi:10.1371/journal.pone.0284029)

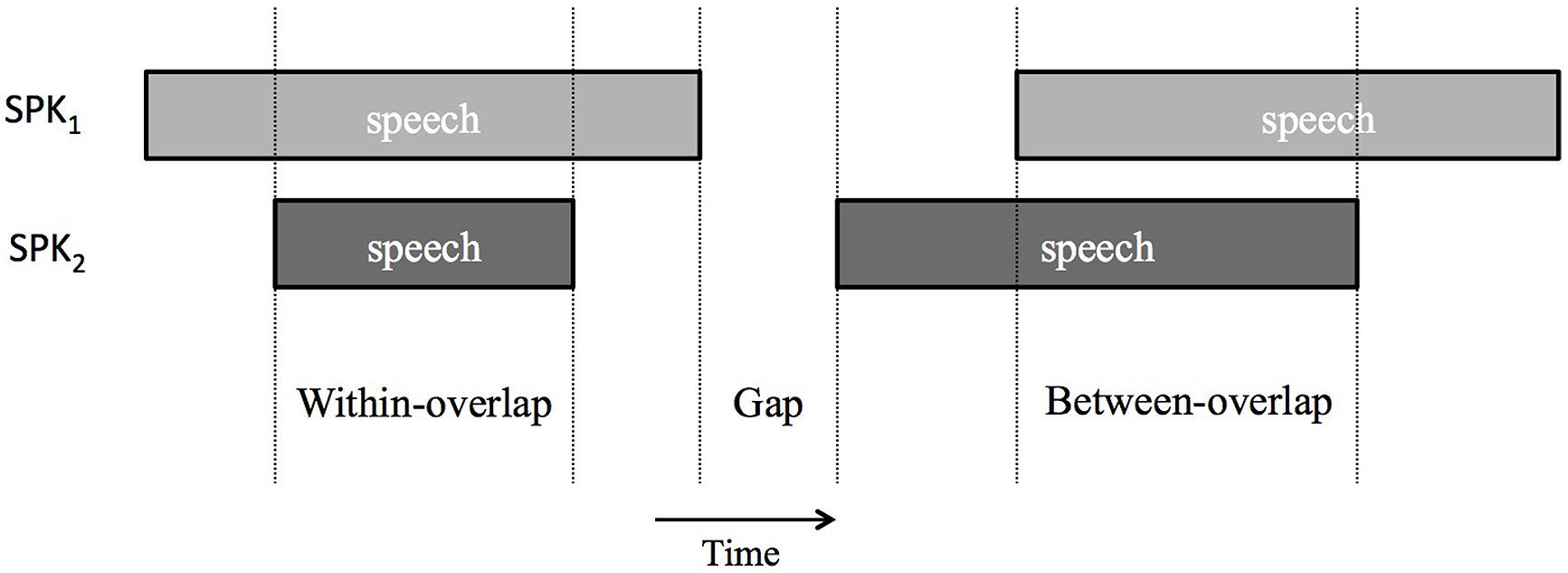

Supplement: S1 Fig — “Gaps” are silent intervals between turn transitions; “between-overlaps” are turn transitions composed of overlapping speech from both interlocutors. “Within-overlaps” are not true floor transfer transitions, but rather represent passages of overlapping speech which are not followed by a change of speaker (and therefore did not enter into turn-timing analyses). Adapted from [24]. (TIF) [file pone.0284029.s002.tif]

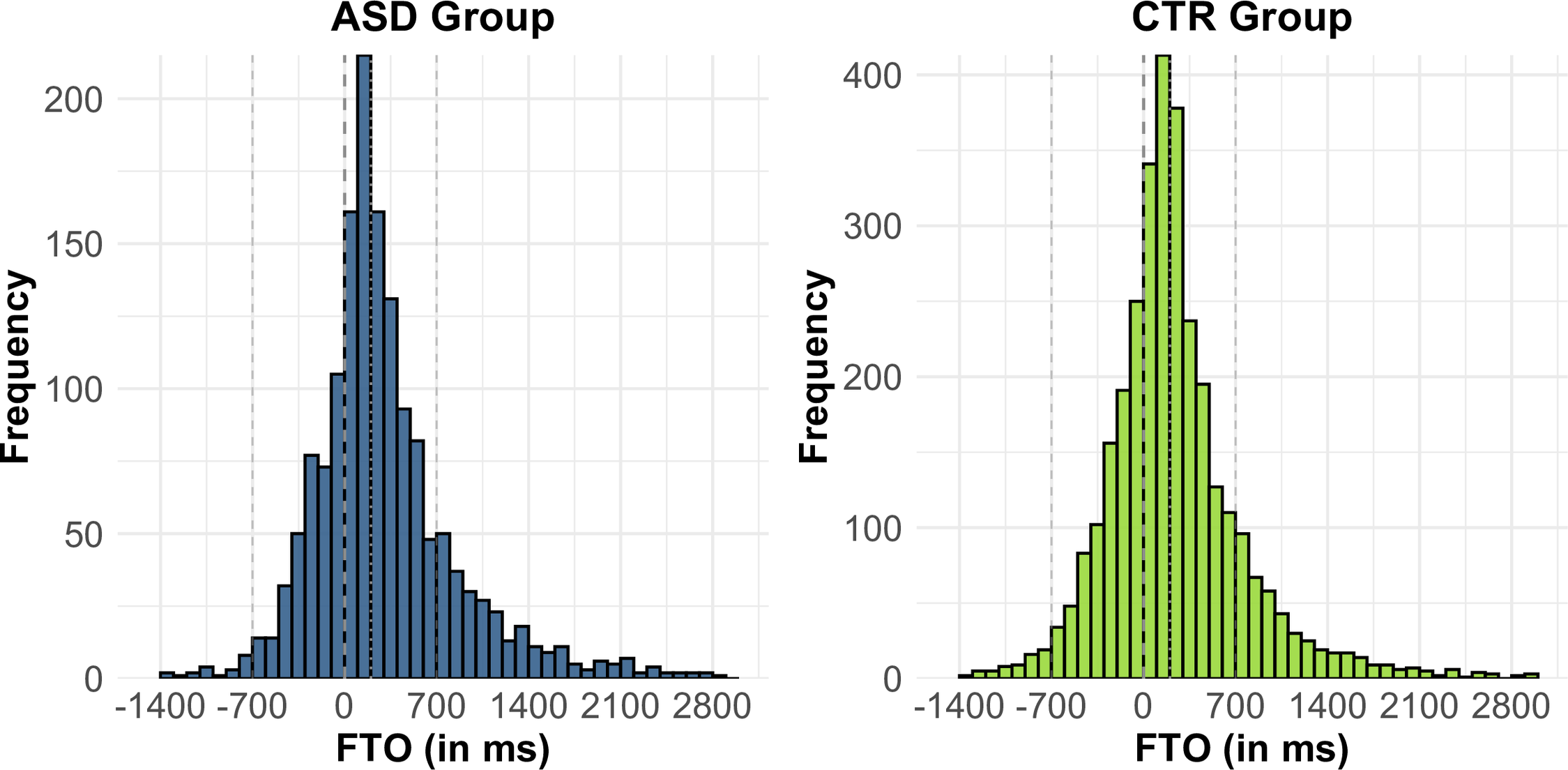

Supplement: S2 Fig — ASD group in the left panel, CTR group in the right panel. Bin width = 100ms. Positive values represent gaps, negative values represent overlaps. (TIF) [file pone.0284029.s003.tif]
